# Supplementary material for: Vegetation Cover Management and Landscape Plant Species Composition Influence the Chrysopidae Community in the Olive Agroecosystem
Source: Plants (Basel). 2022 Nov 27;11(23):3255. doi: 10.3390/plants11233255 (PMC9739066; doi:10.3390/plants11233255)
Supplement: Supplementary file 1 [file plants-11-03255-s001.zip › plants-2003607-supplementary.pdf]

Supplementary Material

Vegetation Cover Management and Landscape Plant Species Composition Influence the Chrysopidae Community in the Olive Agroecosystem

Rafael Alcalá Herrera [1,2,*], Antonio García-Fuentes [3], María Eugenia Ramos-Font [4], Mª Luisa Fernández-Sierra [2] and Francisca Ruano [5]

[1] Department of Agronomy, University of Córdoba, Campus de Rabanales, Building C4 "Celestino Mutis", 14014 Córdoba, Spain
[2] Department of Environmental Protection, Estación Experimental del Zaidín (EEZ-CSIC), C/Profesor Albareda 1, 18008 Granada, Spain
[3] Departamento de Biología Animal, Biología Vegetal y Ecología, Edificio B3, Universidad de Jaén, Campus Las Lagunillas s/n, 23071 Jaén, Spain
[4] Servicio de Evaluación, Restauración y Protección de Agrosistemas Mediterráneos, Estación Experimental del Zaidín (EEZ-CSIC). C/Profesor Albareda 1, 18008 Granada, Spain
[5] Department of Zoology, University of Granada, Campus de Fuentenueva s/n, 18071 Granada, Spain
* Correspondence: a02alher@uco.es; Tel.: +34-637-017-057

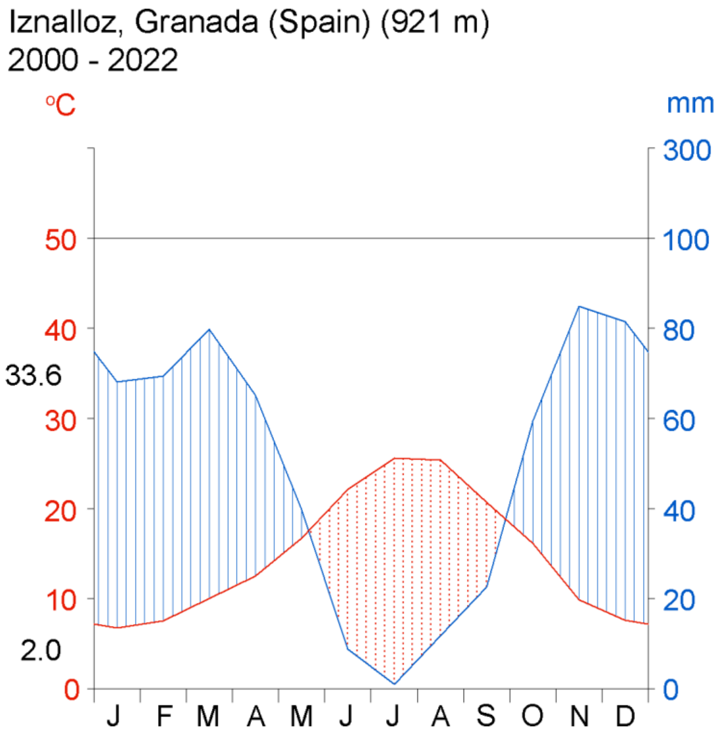

Figure S1: Walther and Lieth climograph from the weather station at Iznalloz (Granada, Spain) between 2000 and 2022. The mean daily maximum and minimum temperatures were 33.6 °C and 2.0 °C, respectively. The annual mean temperature was 15.1 °C and the annual cumulative mean precipitation was 592 mm.

Table S1: Results of the statistical analyses of the flora and vegetation indices.

Sampling	Model	Statistical test	Factor/s	χ^2	d.f.	*P*-value
Cover vegetation	Total plant cover	GLMM (Beta-binomial)	Vegetation cover management	19.87	2	< 0.001
			Transect position	14.01	1	< 0.001
	Cumulative plant cover	GLMM (Beta-binomial)	Vegetation cover management	12.66	2	< 0.01
			Transect position	10.24	1	< 0.01
	Richness	GLMM (Poisson)	Vegetation cover management	8.55	2	< 0.05
			Transect position	0.56	1	0.453
	Diversity	LMM (Gaussian)	Vegetation cover management	6.05	2	< 0.05
			Transect position	0.15	1	0.700
Patch vegetation	Total plant cover	GLMM (Beta-binomial)	Olive farm	6.71	4	0.152
	Cumulative plant cover	GLMM (Beta-binomial)	Olive farm	5.33	4	0.255
	Richness	GLMM (Poisson)	Olive farm	24.01	4	< 0.001
	Diversity	LMM (Gaussian)	Olive farm	6.10	4	0.192

Table S2: Plant cover per species (%) (mean ± SE) for the vegetation cover management and transect position in 2016 and 2017 for the cover vegetation.

Family	Species	2016					2017				
		Vegetation cover management			Transect position		Vegetation cover management			Transect position	
		Grazing	Mowing	Tillage	Beneath the tree canopy	Row	Grazing	Mowing	Tillage	Beneath the tree canopy	Row
Apiaceae	*Torilis arvensis* (Huds.) Link			1.00 ± 0.00		1.00 ± 0.00					
Asteraceae	*Anacyclus clavatus* (Desf.) Pers.		4.29 ± 1.58	28.00 ± 6.05	9.00 ± 3.16	23.88 ± 7.34		8.33 ± 2.29	19.25 ± 3.57	13.50 ± 4.29	11.50 ± 1.71
Asteraceae	*Andryala integrifolia* L.						2.00 ± 0.00	1.00 ± 0.00		1.00 ± 0.00	1.00 ± 0.00
Asteraceae	*Andryala ragusina* L.	1.00 ± 0.00	2.50 ± 1.50			2.00 ± 1.00				2.00 ± 0.00	
Asteraceae	*Anthemis arvensis* L.	4.29 ± 2.00			7.67 ± 4.18	1.75 ± 0.48	5.25 ± 1.84			5.14 ± 2.57	5.40 ± 2.91
Asteraceae	*Calendula arvensis* L.	2.67 ± 1.20	4.67 ± 2.33		6.50 ± 3.28	2.00 ± 0.55	8.00 ± 4.42	4.75 ± 2.01	1.00 ± 0.00	6.69 ± 2.41	2.00 ± 0.52
Asteraceae	*Carduus pycnocephalus* L.						30.00 ± 0.00			30.00 ± 0.00	
Asteraceae	*Centaurea melitensis* L.	1.00 ± 0.00				1.00 ± 0.00	6.00 ± 0.00			6.00 ± 0.00	
Asteraceae	*Chamaemelum fuscatum* (Brot.) Vasc.		4.14 ± 1.18		3.67 ± 1.45	4.50 ± 1.94		4.00 ± 0.58		4.33 ± 0.33	3.75 ± 1.03
Asteraceae	*Chondrilla juncea* L.		5.00 ± 1.73	2.00 ± 0.00	3.50 ± 1.50	5.00 ± 3.00		1.33 ± 0.33	1.00 ± 0.00	1.50 ± 0.50	1.00 ± 0.00
Asteraceae	*Crepis vesicaria* L.	3.00 ± 0.00	13.31 ± 3.28	32.50 ± 8.94	22.18 ± 7.39	17.45 ± 4.41	5.00 ± 4.00	7.62 ± 2.69	21.25 ± 8.26	14.17 ± 3.93	3.43 ± 1.25
Asteraceae	*Filago lutescens* Jord.	2.00 ± 0.00	1.00 ± 0.00			1.50 ± 0.50	1.33 ± 0.33	1.00 ± 0.00		1.00 ± 0.00	1.33 ± 0.33
Asteraceae	*Hedypnois rhagadioloides* (L.) F.W. Schmidt		2.00 ± 0.00	9.38 ± 3.36	3.00 ± 1.35	13.00 ± 4.67			2.00 ± 0.71	2.00 ± 0.71	
Asteraceae	*Hypochaeris glabra* L.	1.00 ± 0.00			1.00 ± 0.00						
Asteraceae	*Lactuca viminea* (L.) J. & C. Presl						1.00 ± 0.00				1.00 ± 0.00
Asteraceae	*Leontodon longirostris* (Finch & P. D. Sell) Talavera	44.88 ± 4.03	8.57 ± 1.58	6.00 ± 3.27	20.67 ± 4.61	27.89 ± 5.70	33.44 ± 4.41	13.31 ± 2.42	3.00 ± 0.58	14.88 ± 2.85	30.93 ± 5.10
Asteraceae	*Scorzonera laciniata* L.		1.67 ± 0.33	1.20 ± 0.20	1.33 ± 0.21	1.62 ± 0.38		8.50 ± 4.75		3.00 ± 0.41	19.50 ± 12.50
Asteraceae	*Senecio vulgaris* L.	1.00 ± 0.00			1.00 ± 0.00		1.00 ± 0.00	1.00 ± 0.00		1.00 ± 0.00	1.00 ± 0.00
Asteraceae	*Sonchus asper* (L.) Hill						5.40 ± 2.48			7.00 ± 4.16	3.00 ± 0.00
Asteraceae	*Sonchus oleraceus* L.			7.38 ± 2.07	10.75 ± 3.40	4.00 ± 0.91			4.00 ± 2.00	4.00 ± 2.00	
Asteraceae	*Taraxacum obovatum* (Willd.) DC.	3.50 ± 0.50	8.00 ± 0.00		3.00 ± 0.00	6.00 ± 2.00					4.00 ± 0.00
Asteraceae	*Tragopogon pratensis* L.						3.00 ± 0.00	4.00 ± 0.00		3.00 ± 0.00	
Asteraceae	*Urospermum picroides* (L.) F. W. Schmidt			1.50 ± 0.50	2.00 ± 0.00	1.00 ± 0.00	1.50 ± 0.50		14.00 ± 0.00	8.00 ± 6.00	1.00 ± 0.00
Boraginaceae	*Nonea micrantha* Boiss. & Reut.						2.00 ± 0.00			2.00 ± 0.00	
Brassicaceae	*Alyssum alyssoides* (L.) L.							6.00 ± 4.51			
Brassicaceae	*Alyssum granatense* Boiss. & Reut.							1.00 ± 0.00			
Brassicaceae	*Alyssum simplex* Rudolphi						1.00 ± 0.00				1.00 ± 0.00
Brassicaceae	*Capsella bursa-pastoris* (L.) Medik.	1.50 ± 0.50	3.00 ± 0.00		1.50 ± 0.50	3.00 ± 0.00	1.00 ± 0.00	1.75 ± 0.48		1.40 ± 0.40	1.50 ± 0.50

Table S2. Continued

Family	Species	2016					2017				
		Vegetation cover management			Transect position		Vegetation cover management			Transect position	
		Grazing	Mowing	Tillage	Beneath the tree canopy	Row	Grazing	Mowing	Tillage	Beneath the tree canopy	Row
Brassicaceae	*Diplotaxis catholica* (L.) DC.	2.00 ± 0.00	4.67 ± 2.33		2.00 ± 0.00	4.67 ± 2.33	7.17 ± 1.83	2.67 ± 0.42		6.43 ± 1.65	2.80 ± 0.86
Brassicaceae	*Diplotaxis erucoides* (L.) DC.			16.40 ± 4.08	17.50 ± 5.07	12.00 ± 0.00					
Brassicaceae	*Diplotaxis viminea* (L.) DC.	2.20 ± 1.20			3.00 ± 2.00	1.00 ± 0.00	3.25 ± 1.03			2.33 ± 0.67	6.00 ± 0.00
Brassicaceae	*Diplotaxis virgata* (Cav.) DC. subsp. *virgata*		3.50 ± 2.50		6.00 ± 0.00	1.00 ± 0.00	4.67 ± 2.73	2.00 ± 0.00		4.00 ± 2.04	
Brassicaceae	*Sisymbrium officinale* (L.) Scop.						1.00 ± 0.00			1.00 ± 0.00	
Brassicaceae	*Thlaspi perfoliatum* L.							2.00 ± 0.00			2.00 ± 0.00
Caryophyllaceae	*Cerastium brachypetalum* Pers. subsp. *brachypetalum*						1.75 ± 0.48			3.00 ± 0.00	1.33 ± 0.33
Caryophyllaceae	*Herniaria cinerea* DC.		1.17 ± 0.17		1.00 ± 0.00	1.50 ± 0.50	7.00 ± 0.00	2.00 ± 0.00		7.00 ± 0.00	2.00 ± 0.00
Caryophyllaceae	*Minuartia hybrida* (Vill.) Schischk. subsp. *hybrida*	5.33 ± 2.45	1.50 ± 0.50		1.67 ± 0.33	6.00 ± 2.88	5.00 ± 1.41	2.50 ± 0.65		5.40 ± 2.54	3.80 ± 1.08
Caryophyllaceae	*Paronychia argentea* Lam.							1.00 ± 0.00			1.00 ± 0.00
Caryophyllaceae	*Petrorhagia nanteuilii* (Burnat) P. W. Ball & Heywood		1.00 ± 0.00		1.00 ± 0.00						
Caryophyllaceae	*Silene diversifolia* Otth						1.00 ± 0.00			1.00 ± 0.00	
Caryophyllaceae	*Silene nocturna* L.		1.00 ± 0.00			1.00 ± 0.00					
Caryophyllaceae	*Silene tridentata* Desf.		1.50 ± 0.50			1.50 ± 0.50					
Caryophyllaceae	*Silene vulgaris* (Moench) Garcke		1.50 ± 0.50	3.00 ± 0.00	1.00 ± 0.00	2.50 ± 0.50					
Caryophyllaceae	*Stellaria pallida* (Dumort.) Piré	3.60 ± 0.87	3.33 ± 0.92		2.80 ± 0.80	4.00 ± 0.89	2.25 ± 1.25	7.38 ± 1.83		8.20 ± 2.30	3.29 ± 1.04
Cistaceae	*Helianthemum angustatum* Pomel							4.00 ± 1.48		1.00 ± 0.00	4.75 ± 1.65
Cistaceae	*Helianthemum salicifolium* (L.) Mill.		1.00 ± 0.00		1.00 ± 0.00						
Convolvulaceae	*Convolvulus arvensis* L.		2.00 ± 0.58	2.00 ± 0.00	2.00 ± 0.00	2.00 ± 1.00		6.00 ± 5.00		6.00 ± 5.00	
Fabaceae	*Astragalus hamosus* L.	2.00 ± 0.58	2.67 ± 0.33	5.57 ± 2.09	4.40 ± 2.42	3.88 ± 1.36		2.00 ± 0.71	4.00 ± 3.00	3.25 ± 2.25	2.33 ± 0.88
Fabaceae	*Astragalus sesameus* L.		2.33 ± 1.15		1.00 ± 0.00	5.00 ± 3.00		2.33 ± 0.88		2.00 ± 0.58	2.60 ± 1.60
Fabaceae	*Bituminaria bituminosa* (L.) C. H. Stirt.						7.00 ± 0.00			7.00 ± 0.00	
Fabaceae	*Coronilla scorpioides* (L.) W. D. J. Koch			1.00 ± 0.00		1.00 ± 0.00	1.00 ± 0.00	1.67 ± 0.67		1.00 ± 0.00	2.00 ± 0.00
Fabaceae	*Medicago littoralis* Loisel.		1.50 ± 0.50		1.00 ± 0.00	2.00 ± 0.00		2.00 ± 0.00			2.00 ± 0.00
Fabaceae	*Medicago minima* (L.) L.	45.25 ± 5.95	7.11 ± 2.83	1.00 ± 0.00	31.27 ± 9.19	27.88 ± 6.40	52.38 ± 6.80	1.80 ± 0.37		39.44 ± 10.07	41.00 ± 10.12
Fabaceae	*Medicago orbicularis* (L.) Bartal.	1.00 ± 0.00	5.50 ± 2.72		1.00 ± 0.00	7.00 ± 3.22		7.00 ± 0.00			7.00 ± 0.00
Fabaceae	*Medicago polymorpha* L.	2.20 ± 0.58	3.60 ± 1.60	14.00 ± 7.57	1.86 ± 0.46	9.67 ± 4.02		11.88 ± 2.51	10.75 ± 5.19	11.25 ± 3.03	12.00 ± 3.70
Fabaceae	*Medicago rigidula* (L.) All.	21.75 ± 4.56	3.17 ± 1.25	6.83 ± 1.40	10.25 ± 4.99	12.67 ± 3.06	6.38 ± 1.93	3.50 ± 0.50	9.25 ± 3.64	7.38 ± 1.93	6.00 ± 2.58
Fabaceae	*Medicago truncatula* Gaertn.	5.00 ± 0.00				5.00 ± 0.00	11.00 ± 1.00	6.43 ± 2.31		5.00 ± 2.65	9.00 ± 2.17

Table S2. Continued

Family	Species	2016					2017				
		Vegetation cover management			Transect position		Vegetation cover management			Transect position	
		Grazing	Mowing	Tillage	Beneath the tree canopy	Row	Grazing	Mowing	Tillage	Beneath the tree canopy	Row
Fabaceae	*Melilotus* Mill.							3.00 ± 0.00			3.00 ± 0.00
Fabaceae	*Trifolium scabrum* L.		6.33 ± 2.72		1.00 ± 0.00	9.00 ± 3.37		3.00 ± 0.00		3.00 ± 0.00	
Fabaceae	*Trifolium tomentosum* L.		11.20 ± 4.09		3.75 ± 1.11	16.17 ± 6.11		13.09 ± 3.25		6.67 ± 2.43	20.80 ± 4.71
Fabaceae	*Trigonella monspeliaca* L.	2.20 ± 0.97	1.40 ± 0.40	2.50 ± 0.87	2.00 ± 0.60	2.00 ± 0.68	1.00 ± 0.00	1.20 ± 0.20		1.00 ± 0.00	1.25 ± 0.25
Fabaceae	*Trigonella polyceratia* L.						2.33 ± 0.67		7.50 ± 2.50	6.00 ± 2.08	2.00 ± 1.00
Fabaceae	*Vicia lutea* L. subsp. lutea	1.00 ± 0.00			1.00 ± 0.00		8.00 ± 0.00			8.00 ± 0.00	
Fabaceae	*Vicia peregrina* L.							2.00 ± 0.00			2.00 ± 0.00
Geraniaceae	*Erodium aethiopicum* (Lam.) Brumh. & Thell.	3.33 ± 0.73	3.75 ± 2.10		2.86 ± 0.91	4.17 ± 1.30				1.50 ± 0.50	3.00 ± 1.08
Geraniaceae	*Erodium ciconium* (L.) L'Hér.						4.64 ± 1.16	11.00 ± 0.00		3.14 ± 1.50	6.75 ± 1.57
Geraniaceae	*Erodium cicutarium* (L.) L'Hér.							3.00 ± 2.31		8.00 ± 2.00	2.00 ± 0.00
Geraniaceae	*Erodium chium* (L.) Willd.			1.00 ± 0.00		1.00 ± 0.00					
Geraniaceae	*Erodium malacoides* (L.) L´Hér. subsp. *malacoides*		22.40 ± 8.98		22.67 ± 14.25	22.00 ± 14.00		4.67 ± 2.03		3.00 ± 2.00	8.00 ± 0.00
Geraniaceae	*Erodium primulaceum* (Lange) Lange		12.17 ± 4.77		8.00 ± 7.00	14.25 ± 6.66		4.17 ± 1.91		3.33 ± 1.20	5.00 ± 4.00
Geraniaceae	*Geranium molle* L.	13.56 ± 3.16	9.62 ± 2.56		9.08 ± 2.53	13.80 ± 3.08	19.78 ± 6.29	7.33 ± 1.41	1.00 ± 0.00	14.62 ± 4.69	8.25 ± 2.08
Lamiaceae	*Lamium amplexicaule* L.							3.00 ± 0.00		3.00 ± 0.00	
Malvaceae	*Malva cretica* Cav. subsp. *althaeoides* (Cav.) Dalby		5.80 ± 1.86		8.33 ± 1.76	2.00 ± 1.00		1.00 ± 0.00			1.00 ± 0.00
Malvaceae	*Malva neglecta Wallr.*			1.00 ± 0.00	1.00 ± 0.00						
Malvaceae	*Malva nicaensis* All.		10.67 ± 5.55		10.67 ± 5.55			30.00 ± 7.45		30.00 ± 7.45	
Papaveraceae	*Papaver rhoeas* L.	1.00 ± 0.00			1.00 ± 0.00						
Plantaginaceae	*Plantago albicans* L.							1.67 ± 0.67		1.00 ± 0.00	2.00 ± 1.00
Plantaginaceae	*Plantago lagopus* L.			3.33 ± 2.33	1.00 ± 0.00	8.00 ± 0.00			16.00 ± 12.50	16.00 ± 12.50	
Plantaginaceae	*Plantago lanceolata* L.			29.75 ± 16.71	5.00 ± 0.00	38.00 ± 20.55					
Plantaginaceae	*Veronica agrestis* L.		3.00 ± 1.53		4.00 ± 2.00	1.00 ± 0.00		2.50 ± 0.50			2.50 ± 0.50
Plantaginaceae	*Veronica polita* Fr.		1.00 ± 0.00			1.00 ± 0.00		1.00 ± 0.00			1.00 ± 0.00
Poaceae	*Aira caryophyllea* L.		1.00 ± 0.00		1.00 ± 0.00			6.00 ± 4.51		9.00 ± 7.51	1.50 ± 0.50
Poaceae	*Avena barbata* Link	3.67 ± 1.20		8.50 ± 3.50	6.00 ± 0.00	5.50 ± 2.26	13.00 ± 0.00			13.00 ± 0.00	
Poaceae	*Avena sterilis* L.			1.75 ± 0.48	1.40 ± 0.25	3.00 ± 0.00		1.00 ± 0.00	1.00 ± 0.00	1.00 ± 0.00	
Poaceae	*Bromus diandrus* Roth		1.00 ± 0.00		1.00 ± 0.00			2.60 ± 1.17		4.00 ± 3.00	1.67 ± 0.67

Table S2. Continued

Family	Species	2016					2017				
		Vegetation cover management			Transect position		Vegetation cover management			Transect position	
		Grazing	Mowing	Tillage	Beneath the tree canopy	Row	Grazing	Mowing	Tillage	Beneath the tree canopy	Row
Poaceae	*Bromus fasciculatus* C. Presl subsp. *fasciculatus*						6.08 ± 1.25			7.33 ± 2.16	4.83 ± 1.25
Poaceae	*Bromus hordeaceus* L.	1.00 ± 0.00	3.00 ± 0.00	5.67 ± 4.18	1.33 ± 0.33	8.50 ± 5.50		15.25 ± 4.21	7.50 ± 4.72	10.00 ± 3.53	15.50 ± 9.50
Poaceae	*Bromus rigidus* Roth							3.00 ± 0.00	2.00 ± 0.00	2.00 ± 0.00	3.00 ± 0.00
Poaceae	*Bromus rubens* L. subsp. *rubens*	4.00 ± 1.01		4.00 ± 3.00	4.67 ± 1.61	3.33 ± 0.96		1.00 ± 0.00		1.00 ± 0.00	
Poaceae	*Bromus sterilis* L.	5.75 ± 3.54	1.00 ± 0.00		3.00 ± 2.00	4.75 ± 3.75	7.00 ± 6.00			7.00 ± 6.00	
Poaceae	*Bromus tectorum* L.						1.00 ± 0.00				1.00 ± 0.00
Poaceae	*Catapodium rigidum* (L.) C. E. Hubb. subsp. *rigidum*		4.11 ± 1.40		2.75 ± 1.75	5.20 ± 2.13					
Poaceae	*Cynodon dactylon* (L.) Pers.		1.00 ± 0.00	14.00 ± 0.00	1.00 ± 0.00	14.00 ± 0.00					
Poaceae	*Echinaria capitata* (L.) Desf.	2.00 ± 0.00			2.00 ± 0.00		1.33 ± 0.33			1.00 ± 0.00	2.00 ± 0.00
Poaceae	*Hordeum murinum* L. subsp. *leporinum*	9.25 ± 8.25	5.79 ± 1.87	1.33 ± 0.33	9.45 ± 3.36	1.80 ± 0.39	4.17 ± 1.35	8.64 ± 1.54	12.00 ± 7.55	10.00 ± 1.86	4.00 ± 1.00
Poaceae	*Lolium rigidum* Gaudin			4.17 ± 1.14	3.75 ± 1.75	5.00 ± 0.00					
Poaceae	*Micropyrum tenellum* (L.) Link		1.00 ± 0.00		1.00 ± 0.00			1.00 ± 0.00			1.00 ± 0.00
Poaceae	*Mibora minima* (L.) Desv.							1.00 ± 0.00			1.00 ± 0.00
Poaceae	*Poa annua* L.		2.00 ± 0.00			2.00 ± 0.00					
Poaceae	*Poa bulbosa* L.		1.00 ± 0.00		1.00 ± 0.00			4.50 ± 0.50		4.00 ± 0.00	5.00 ± 0.00
Poaceae	*Rostraria cristata* (L.) Tzvelev		9.33 ± 3.89		2.67 ± 0.33	16.00 ± 5.57		18.43 ± 5.11		8.83 ± 4.22	25.62 ± 7.62
Poaceae	*Vulpia ciliata* Dumort. subsp. *ciliata*		4.50 ± 3.50			4.50 ± 3.50		4.50 ± 2.87		1.00 ± 0.00	8.00 ± 5.00
Poaceae	*Vulpia myuros* (L.) C. C. Gmel.	1.00 ± 0.00				1.00 ± 0.00	2.40 ± 0.60	6.20 ± 1.83		2.80 ± 1.11	5.80 ± 1.77
Poaceae	*Vulpia unilateralis* (L.) Stace		2.83 ± 0.70		1.67 ± 0.33	4.00 ± 1.00		19.12 ± 2.25		17.75 ± 3.95	20.50 ± 2.60
Primulaceae	*Anagallis foemina* Mill.						1.00 ± 0.00			1.00 ± 0.00	
Primulaceae	*Asterolinon linum–stellatum* (L.) Duby						2.00 ± 0.00				2.00 ± 0.00
Rosaceae	*Sanguisorba verrucosa* (G. Don) Ces.							1.00 ± 0.00		1.00 ± 0.00	
Rubiaceae	*Callipeltis cucullaris* (L.) Steven		3.50 ± 0.50			3.50 ± 0.50					
Rubiaceae	*Galium aparine* L.	3.00 ± 0.00	1.00 ± 0.00		1.00 ± 0.00	2.00 ± 1.00	3.25 ± 1.44		4.00 ± 0.00	4.00 ± 1.73	1.00 ± 0.00
Rubiaceae	*Galium murale* (L.) All.	1.00 ± 0.00	5.00 ± 0.00		1.00 ± 0.00	5.00 ± 0.00		1.50 ± 0.29		1.50 ± 0.50	1.50 ± 0.50
Rubiaceae	*Galium parisiense* L. subsp. *parisiense*	2.00 ± 0.00			2.00 ± 0.00		2.00 ± 0.00			2.67 ± 0.67	
Rubiaceae	*Galium verticillatum* Lam.		2.60 ± 0.68	10.00 ± 4.93	11.50 ± 7.50	3.33 ± 1.23	3.50 ± 1.50	3.00 ± 1.53	11.50 ± 9.50	8.75 ± 4.21	2.20 ± 0.97
Urticaceae	*Urtica urens* L.							3.00 ± 0.00		3.00 ± 0.00	
Veronicaceae	*Veronica hederifolia* L.						1.00 ± 0.00			1.00 ± 0.00	

Table S3: Plant cover per species (%) (mean ± SE) in each olive farm for the patch vegetation.

Family	Species	Olive farm				
		**La Pedriza**	**Los Almendros**	**Norberto**	**Píñar (right)**	**Píñar (left)**
Anacardiaceae	*Pistacia lentiscus* L.	0.04 ± 0.00				
Anacardiaceae	*Pistacia terebinthus* L.	4.80 ± 0.00	9.10 ± 5.27			
Apiaceae	*Foeniculum vulgare* Mill.		2.60 ± 0.00			
Asparagaceae	*Asparagus acutifolius* L.		2.15 ± 1.16			
Asteraceae	*Helichrysum stoechas* (L.) Moench		0.60 ± 0.20			
Asteraceae	*Staehelina dubia* L.		0.60 ± 0.20	0.80 ± 0.00		
Boraginaceae	*Lithodora fruticosa* (L.) Griseb.				2.30 ± 0.30	
Caryophyllaceae	*Silene vulgaris* (Moench) Garcke		0.40 ± 0.00	4.20 ± 0.00		
Cistaceae	*Cistus albidus* L.	8.88 ± 3.26		23.20 ± 3.08		
Cistaceae	*Cistus clusii* Dunal			2.70 ± 0.10	1.40 ± 0.00	7.13 ± 4.34
Cistaceae	*Fumana thymifolia* (L.) Webb		2.50 ± 1.20	2.47 ± 0.37		
Cistaceae	*Helianthemum cinereum* (Cav.) Pers. subsp. *rotundifolium*		1.44 ± 0.41		2.80 ± 1.51	
Cistaceae	*Helianthemum hirtum* (L.) Mill.			4.00 ± 0.00		
Cupressaceae	*Juniperus oxycedrus* L.	4.11 ± 1.52		12.60 ± 0.00	11.20 ± 1.40	
Cyperaceae	*Carex hallerana* Asso	0.60 ± 0.00			0.80 ± 0.00	
Fabaceae	*Argyrolobium zanonii* (Turra) P.W. Ball subsp. *zanonii*	0.04 ± 0.00	0.40 ± 0.00			
Fabaceae	*Astragalus incanus* L. subsp. *nummularioides*	1.40 ± 0.00	1.10 ± 0.50			
Fabaceae	*Genista cinerea* (Vill.) DC. subsp. *cinerea*		35.40 ± 12.33	3.20 ± 0.00	6.80 ± 0.00	
Fabaceae	*Genista scorpius* (L.) DC.				2.20 ± 0.00	2.30 ± 0.10
Fabaceae	*Retama sphaerocarpa* (L.) Boiss.			1.40 ± 0.00		
Fabaceae	*Ulex parviflorus* Pourr.		11.47 ± 1.27	7.53 ± 5.67	3.00 ± 0.00	
Fagaceae	*Quercus coccifera* L.	10.00 ± 2.42	13.52 ± 4.61		24.00 ± 8.20	5.20 ± 0.80
Fagaceae	*Quercus faginea* Lam. subsp. *faginea*		16.40 ± 0.00			
Fagaceae	*Quercus rotundifolia* Lam.	12.44 ± 3.60	52.84 ± 6.65	7.60 ± 3.60	43.66 ± 6.21	50.78 ± 10.52

Table S3. Continued

Family	Species	Olive farm				
		**La Pedriza**	**Los Almendros**	**Norberto**	**Píñar (right)**	**Píñar (left)**
Lamiaceae	*Lavandula latifolia* Medik.		0.60 ± 0.00		8.60 ± 0.00	
Lamiaceae	*Phlomis lychnitis* L.			1.80 ± 0.00	1.50 ± 0.90	
Lamiaceae	*Rosmarinus officinalis* L.	7.10 ± 2.56		13.20 ± 2.80	14.07 ± 3.12	20.37 ± 5.87
Lamiaceae	*Teucrium pseudochamaepitys* L.	1.92 ± 0.57	1.00 ± 0.35	0.60 ± 0.00	0.70 ± 0.10	2.20 ± 0.00
Lamiaceae	*Thymus mastichina* (L.) L. subsp. *mastichina*			2.50 ± 0.50	0.60 ± 0.00	
Lamiaceae	*Thymus zygis* L. subsp. *gracilis* (Boiss.) R. Morales	2.60 ± 0.00	4.20 ± 1.00	3.33 ± 1.55	1.47 ± 0.64	10.13 ± 6.64
Linaceae	*Linum suffruticosum* L.			0.60 ± 0.00		
Oleaceae	*Olea europaea* L.	0.60 ± 0.00		0.60 ± 0.00		
Pinaceae	*Pinus halepensis* Mill.	33.86 ± 8.57		49.00 ± 0.00	37.40 ± 3.65	
Pinaceae	*Pinus pinaster* Aiton			10.40 ± 0.00		
Poaceae	*Brachypodium retusum* (Pers.) P. Beauv. subsp. *retusum*	10.56 ± 2.26	21.96 ± 8.02	8.00 ± 0.00	10.74 ± 2.77	9.60 ± 0.20
Poaceae	*Dactylis glomerata* L. subsp. *hispanica*	4.56 ± 2.03	4.85 ± 1.49	0.40 ± 0.00	0.40 ± 0.00	
Poaceae	*Macrochloa tenacissima* (L.) Kunth	13.85 ± 9.45	13.50 ± 12.90	15.40 ± 0.80	18.70 ± 8.21	32.60 ± 8.90
Poaceae	*Piptatherum miliaceum* (L.) Coss.		7.40 ± 0.00	1.50 ± 1.10	5.40 ± 0.00	
Rhamnaceae	*Rhamnus lycioides* L.	2.40 ± 0.00	17.07 ± 7.00			8.60 ± 0.00
Rosaceae	*Prunus dulcis* (Mill.) D. A. Webb		66.60 ± 19.23	55.73 ± 4.58		
Rubiaceae	*Rubia peregrina* L.		0.40 ± 0.00			
Thymelaeaceae	*Daphne gnidium* L.		1.20 ± 0.00	2.50 ± 1.10		
